# Leaf Concentrate Fortification of Antenatal Protein-Calorie Snacks Improves Pregnancy Outcomes

**Published:** 2014-09

**Authors:** Anjna Magon, Simon M. Collin, Pallavi Joshi, Glyn Davys (Late), Amita Attlee, Beena Mathur

**Affiliations:** ^1^Department of Foods and Nutrition, University of Rajasthan, Jaipur, India; ^2^School of Social & Community Medicine, University of Bristol, UK; ^3^Association pour la Promotion des Extraits Foliaires en Nutrition (APEF), Nozet, F-51230, Connantre, France; ^4^Department of Clinical Nutrition and Dietetics, College of Health Sciences, University of Sharjah, United Arab Emirates

**Keywords:** Anaemia, Birthweight, Leaf concentrate, Pregnancy, India

## Abstract

Ready-to-eat (RTE) snacks are routinely distributed to pregnant women in India. These provide protein and calories but are low in micronutrients. We investigated whether RTE snacks fortified with leaf concentrate (LC) could improve pregnancy outcomes, including maternal haemoglobin (Hb) concentrations and infants’ birthweight. This randomized controlled two-arm trial was conducted over 18 months: control (sRTE) group received standard 120 g RTE snack (102 g wheat flour, 18 g soya flour); intervention (lcRTE) group received the same snack fortified with 7 g LC. The study was conducted in Jaipur, Rajasthan, India. One hundred and five pregnant women aged 18-35 years were studied. Among the 105 women randomized to the two arms of the trial, 2 (1.9%) were severely anaemic (Hb ≤6.0 g/dL); 55 (53.4%) were moderately anaemic (Hb 6.0-8.0 g/dL); 34 (33.0%) were mildly anaemic (Hb 8.6-10.9 g/dL); and 12 (11.7%) were not anaemic (Hb ≥11.0 g/dL). In the final month of pregnancy, 83.0% (39/47) of women in the sRTE group had Hb ≤8.5 g/dL compared to 37.8% (17/45) in the lcRTE group (p<0.001). After adjustment for age and baseline Hb concentration, the difference in Hb concentrations due to LC fortification was 0.94 g/dL (95% CI 6.8-12.0; p<0.001). Mean live birthweight in the lcRTE group was 2,695 g (SD 325 g) compared to 2,545 g (297 g) in the sRTE group (p=0.02). The lcRTE snacks increased infants’ birthweight by 133.7 g (95% CI 7.3-260.2; p=0.04) compared to sRTE snacks. Leaf concentrate fortification of antenatal protein-calorie snacks in a low-income setting in India protected against declining maternal haemoglobin concentrations and increased infants’ birthweight when compared with unfortified snacks. These findings require replication in a larger trial.

## INTRODUCTION

Malnutrition during pregnancy is a risk factor of maternal anaemia and adverse pregnancy outcomes, including preterm delivery, low birthweight, and neural tube defects (1-3). Studies in India have shown a consistently high (80%) prevalence of anaemia among women in rural and urban districts (4-6).

Antenatal iron and folic acid (IFA) supplements and protein-calorie supplements in the form of ready-to-eat (RTE) snacks or freshly-prepared food are provided to pregnant women in India through the Integrated Child Development Services (ICDS) Programme (7). However, although 65% of mothers received IFA supplements, only 23% of women consumed these supplements for at least 90 days (8). This low level of adherence may be due to the frequent gastrointestinal side-effects of iron supplements (9). Hence, food-based approaches to combating maternal malnutrition may be more effective (6,10), including low-cost locally-produced food-based micronutrient supplements (11). Leaf concentrate (LC) was produced in France in the 18th century and developed as a foodstuff in England between 1940 and 1970 (12). LC has since been promoted by several non-governmental organizations, including Find Your Feet in the UK, Leaf for Life in the USA, and the Association pour la Promotion des Extraits Foliaires en Nutrition (APEF) in France, as a sustainable form of protein and micronutrient supplementation in low-income communities (13,14).

In a previous study, we demonstrated that LC was an effective and more palatable alternative to IFA supplements for treating anaemia in adolescent girls in a low-income urban community in Jaipur (15). Here, we investigate, in the same setting, whether RTE protein-calorie snacks for pregnant women are more effective in preventing maternal anaemia and infants’ low birthweight when fortified with locally-produced LC.

## MATERIALS AND METHODS

### Study population

Our target population comprised pregnant women aged 18-35 years living in a low-income area of Jaipur. The study area was selected by randomly sampling 1 of 2 Integrated Child Development Services (ICDS) blocks, then 1 of 4 *Parikshetras* (districts) in the selected block, then 8 of 24 *Anganwadis* in the selected *Parikshetra*. An *Anganwadi* is a government-sponsored centre that provides care to women and children (aged 0-6 years), including antenatal and postnatal care, immunization, and supplementary nutrition. The selected *Anganwadis* provided services to two slums (Tata Nagar and Shivaji Nagar) with a combined population of approximately 8,000. Pregnant women were identified by means of *Anganwadi* workers making door-to-door visits, accompanied by the field researcher and/or two field assistants. The objectives of the study were explained by the *Anganwadi* workers, and women were recruited according to the following eligibility criteria: early 2nd trimester (14th-16th week of pregnancy), age 18-35 years, gravidity 1-4, expected to be resident in the locality throughout their pregnancy, and delivery to be conducted locally. Women who consented to participate were followed prospectively until full term. The study was conducted between March 1994 and October 1995.

### Study design

We aimed to achieve a sample-size of 50 women in each arm of the trial, corresponding to 90% power to detect a 12% difference (between and within groups) in mean Hb (from 8.5 g/dL to 7.5 g/dL, standard deviation 1.5 g/dL for both measurements) at 5% level of significance. Women were allocated at random to receive either the standard (sRTE) or the LC-fortified (lcRTE) snack once daily. Randomization was done as preset in consecutively-numbered sealed envelopes, each containing a paper slip indicating allocation to lcRTE or sRTE. One envelope was opened by the field researcher each time that a woman consented to participate. Participants were not blinded to their allocation because of the green colour of the lcRTE snacks. A questionnaire was administered by a researcher or field assistant at recruitment to collect data on demographic and socioeconomic status (composition of household, income, assets, education, occupation), dietary patterns (food frequency, pregnancy-related dietary changes, and 24-hour dietary recall), use of ICDS services, and obstetric history; 24-hour dietary recall was repeated twice in the 3rd trimester (at the beginning of the 7th and 9th month of pregnancy). Daily nutrient intakes were estimated using standardized utensils and food compositions (16). Mother's weight was measured monthly from recruitment to delivery. Haemoglobin (Hb) was measured at recruitment (14th-16th week) and at the beginning of the final month of pregnancy (35th or 36th week). Mothers were followed (by means of visits of field-level domiciliary workers) up to delivery and pregnancy outcomes obtained by questionnaire. Infants were weighed 7 days after birth.

### Primary and secondary outcomes

The primary outcomes of our study were maternal haemoglobin concentrations in the 3rd trimester and infants’ birthweight (at delivery). Our secondary outcomes were: the proportion of women with 3rd trimester anaemia (Hb <8.5 g/dL), maternal weight gain during pregnancy (calculated at the end of the 2nd and 3rd trimester), and gestational age (weeks since last menstrual period).

### Standard and fortified RTE snack

The sRTE snack (120 g) was extruded from a 6:1 (by weight) mix of wholemeal wheat (85 g per 100 g) and soya (15 g per 100 g) flour. The lcRTE snack had 6 g (per 100 g) of LC substituted for the same weight of wheat flour. LC from Berseem (*Trifolium alexandrinum*) was supplied in dried powdered form by the Society for the Development of Appropriate Technologies (SOTEC), Bareilly, Uttar Pradesh. The LC powder was analyzed for *Salmonella*, *E. coli,* and total bacterial counts at Saras Sankul (Rajasthan Cooperative of Dairy Federation, Jaipur) before being sent to Modern Food Industries, Jaipur, for incorporation in the fortified RTE snack. The snack was manufactured in monthly batches and stored in airtight food containers at the *Anganwadis*. The nutritional composition of the standard and fortified snacks is shown in Table 1. During the first 2-3 weeks of the study, RTE snack was distributed to the home of each participant on a weekly basis. After this, women would collect a weekly supply from their nearest *Anganwadi* centre. Consumption of RTE snacks was monitored by the field researcher and her assistants through home visits.

### Haemoglobin measurement

The field researcher and both field assistants were trained to measure Hb concentrations at Santokba Durlabhji Memorial Hospital (Department of Pathology), Jaipur. Hb was measured from a 0.02 mL venous blood sample which was transferred to Whatman No. 1 filter paper, air-dried, and kept in a sealed envelope. Every three days in the laboratory of the Department of Home Science, samples were eluted in 5 mL of Drabkins solution (0.05 g potassium cyanide, 0.2 g potassium ferricyanide, 0.14 g dihydrogen potassium phosphate, dissolved in 200-300 mL of water and diluted to 1 L using water plus 1 mL of non-ionic detergent) for 2 hours. The eluted sample was vortexed for 1 minute, and Hb concentration was then measured by colorimetry at 540 nm, using Drabkins solution as control (17).

### Anthropometric measurements

Women were weighed using an adult platform-weighing balance selected for portability and ease-of-use during domiciliary visits. The scales had a sensitivity of 0.5 kg and were calibrated periodically, using a set of 20 kg weights. Women were weighed with clothe (except for shoes or heavy clothing, e.g. woollen garments). Height was measured using a non-flexible metal tape held vertically against a smooth wall against which women were asked to stand with bare feet flat on the floor, heels together, legs straight, and shoulders relaxed. A ruler was held from the top of the woman's head to the tape measure.

### Data analyses

Data were entered into a computer, using Microsoft Excel and were analyzed using Stata Release 12 (StataCorp. 2011. Stata Statistical Software: Release 12. College Station, TX, USA). Differences between mean values of continuous variables were tested using Student's *t*-test, and differences between proportions were tested using the chi-square test or Fisher's exact test (if n <10 in any cell of table). We used three multivariable models to estimate the effect of LC fortification on our primary outcomes: (i) Hb in the 3rd trimester as dependent variable, baseline Hb, mother's age, vegetarianism, education (none vs any) as independent variables; (ii) maternal weight in the 3rd trimester as dependent variable, baseline maternal weight, mother's age, vegetarianism, education, infant's sex, and gestational age (>37 vs ≤37 weeks) as independent variables; (iii) infant's birth weight as dependent variable, mother's age, vegetarianism, education, body mass index (at baseline), child's sex, and gestational age as independent variables.

### Ethical approval

The study was approved by the Ethical Committee of the Department of Home Science, University of Rajasthan, Jaipur. Permission to carry out the study at ICDS *Anganwadis* was obtained from the Department of Women and Child Development, Government of Rajasthan. Written informed consent was obtained from each participant.

**Table 1.** Composition (per 120 g snack) of ready-to-eat snacks: leaf concentrate fortified (lcRTE) and standard (sRTE)*

Nutrient	lcRTE snack [Weight (% of RDA)]†	sRTE snack [Weight (% of RDA)]†
Protein (g)	23.6 (36.3)	20.1 (30.9)
Fat (g)	3.2 (10.7)	1.7 (5.7)
Carbohydrate (g)	69.7	74.5
Energy (kcal)	426 (78.8)	426 (78.8)
Calcium (mg)	223 (23.3)	92 (9.2)
Iron (mg)	13.6 (35.8)	6.9 (18.2)
β-carotene (µg)	1138 (47.4)	106 (4.4)
Folate (µg)	75.7 (18.9)	54.5 (13.6)

*120 g sRTE snack comprised 85 g wheat flour, and 15 g soya flour per 100 g; the 120 g lcRTE snack comprised 79 g wheat flour, 15 g soya flour, and 6 g leaf concentrate per 100 g;

†Recommended Daily Allowance (RDA) for Indians (18) (β-carotene as retinol, 6:1 equivalence)

## RESULTS

### Characteristics of trial participants

One hundred and five women participated in the trial (Figure). The study was conducted in an area of low socioeconomic status (mean per-capita monthly income approximately US$ 67). The average household comprised 6 family members living in mainly single-room *pucca* (brick and stone construction) or semi-*pucca* (brick and mud construction) houses. The characteristics of the women in the two arms of the trial are summarized in Table 2. More than half (55.2%) of the women had no formal education. The mean age of the participants was 23.5 (SD 4.0) years. Mean ages at menarche, marriage, and first pregnancy were 14.5 (1.7), 16.0 (2.5), and 18.5 (2.5) years respectively. One-fifth (21.0%) of women were primigravid; the same proportion was secundigravid; and 15.2% and 42.8% were pregnant for the 3rd and 4th time respectively. One-third (28.6%) of non-primigravid women were pregnant within 12 months of the birth of their previous child. None of the above characteristics or experience of previous miscarriage/stillbirth differed between the two trial groups (Table 2).

### Dietary intake at recruitment (4th month of pregnancy)

Half of the women (47.6%) were vegetarian (although non-vegetarian women consumed very little animal protein due to its cost), and the majority (86.7%) consumed two meals per day during pregnancy. One-fifth (20.9%) of the women reported geophagy, which included consumption of coal, clay, chalk, brick, and lime. These characteristics did not differ between the two groups (Table 2). Mean daily energy intake for participants in both the groups was below the Recommended Daily Allowance for Indians [RDA, Indian Council of Medical Research (18)] for pregnant women (2,175 kcal/day): 1,715 kcal/day (79%) in the lcRTE group, 1,610 kcal/day (74%) in the sRTE group (Table 3). Protein intake was also below the RDA for pregnant women (65 g/day): 50 g/day (77%) in the lcRTE group; 51 g/day (78%) in the sRTE group. In the lcRTE group, intakes of micronutrients as a proportion of RDA were: iron 39%, folate 48%, calcium 47%, β-carotene 12% (as retinol, 6:1 equivalence), vitamin B_12_ 56%, and vitamin C 142%. In the sRTE group, the corresponding proportions were: iron 44%, folate 43%, calcium 53%, β-carotene 16%, vitamin B_12_ 82%, and vitamin C 142% (Table 3). The high vitamin C intake was attributed to the use of tomatoes and lemons in cooking.

### Adherence to RTE snack regimen

The first week's supply of RTE snacks was delivered to women in the 14th-16th week of their pregnancy. The majority of women (91.5% in the lcRTE group, 80.4% in the sRTE group) consumed a daily RTE snack for >20 weeks. Women who reported sharing their snack with their children or other family members were given additional packets of snacks.

**Figure.**
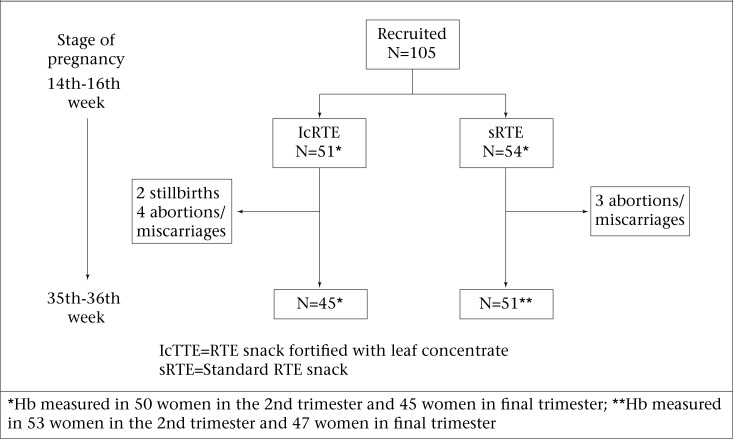
Allocation to trial arms and numbers of participants followed up

**Table 2.** Characteristics of women in each arm of the study at baseline

Characteristics	lcRTE (N=51)		sRTE (N=54)		p value*
	Mean	SD	Mean	SD
Monthly household income (Rupees)	3,026	2,041	3,003	1,676	0.95
Age (years)	23.6	3.42	22.9	3.26	0.23
Age at menarche (years)	14.7	1.68	14.3	1.81	0.26
Age at marriage (years)	15.8	2.50	16.2	2.61	0.47
Age at 1st pregnancy (years)	18.6	2.63	18.4	2.33	0.68
Body mass index (kg/m^2^)	18.7	2.3	18.5	2.3	0.42
Gravidity	n	%	n	%	
1	10	19.6	15	27.8	0.21
2	8	15.7	15	27.8	
3	15	29.4	10	18.5	
4	18	35.3	14	25.9	
Birth interval (months)					
≤12	12	23.5	18	33.3	0.54
13-24	25	49.0	23	42.6	
≥25	14	27.4	13	24.1	
Previous miscarriage/stillbirth	12	23.5	12	22.2	0.87
3 meals per day during pregnancy†	6	11.8	8	14.8	0.65
Vegetarian†	24	47.1	26	48.2	0.91
Geophagy during pregnancy	10	19.6	12	22.2	0.74
No formal education vs some education	27	52.9	32	59.3	0.51
Anaemic (Hb <8.5 g/dL)	24	48.0	22	41.5	0.51

*Student's *t-*test for continuous variables, chi-square test for categorical variables;

†Majority of women ate 2 meals per day; non-vegetarian women consumed very little animal protein due to its cost

### Dietary intake in the 7th and 9th month of pregnancy

Mean daily energy intake for participants increased in both the groups as did mean daily protein intake, by amounts corresponding to the protein/calorie content of the RTE snack (Table 3). Intake of iron, folate, β-carotene (as retinol, 6:1 equivalence), and calcium increased in the lcRTE group to 75%, 60%, 78%, and 68% of RDA respectively in the final month of pregnancy whereas women in the sRTE group received 55%, 54%, 16%, and 58% of RDA of these micronutrients. Fat intake was uniformly high due to extensive use of vegetable oil in cooking, and vitamin C intake exceeded RDA throughout pregnancy due to the use of tomatoes and lemons as ingredients. Throughout our study, iron and folic acid supplements were not available through the ICDS programme due to the shortage of supply.

### Baseline and the 3rd trimester haemoglobin concentrations and anaemia

Among the 105 women randomized to the two arms of the trial: 2 (1.9%) were severely anaemic (Hb ≤6.0 g/dL); 55 (53.4%) were moderately anaemic (Hb 6.0-8.5 g/dL); 34 (33.0%) were mildly anaemic (Hb 8.6-10.9 g/dL); and 12 (11.7%) were not anaemic (Hb ≥11.0 g/dL). There was no difference in the distribution of Hb concentrations in the two groups at recruitment (p=0.81 in Fisher's exact test) and no difference in Hb concentrations comparing vegetarian with non-vegetarian women (p=0.15 in Student's *t-*test). In the final month of pregnancy, 83.0% (39/47) of women in the sRTE group had Hb ≤8.5 g/dL compared to 37.8% (17/45) in the lcRTE group (p<0.001 in chi-square test). There was a mean increase in Hb concentrations of 0.27 g/dL (95% CI 0.08-0.46; p=0.006) in the lcRTE group whereas Hb concentrations fell by 0.59 g/dL (95% CI 0.35-0.82; p<0.001) in the sRTE group (Table 4). In a multivariable linear regression model among mothers with infants born alive, adjusting for baseline Hb concentrations, age, vegetarianism, and education (none vs any), the difference in Hb concentrations due to LC fortification was 0.94 g/dL (95% CI 6.8-12.0; p<0.001).

**Table 3.** Dietary intake in the 4th, 7th, and 9th month of pregnancy by study group [mean (SD), % of RDA‡]

Nutrient	4th month (before supplementation)			7th month (including supplements)		9th month (including supplements)	
	RDA	lcRTE (N=51)	sRTE (N=54)	lcRTE (N=47)	sRTE (N=51)	lcRTE (N=47)	sRTE (N=51)
Protein (g)	65	50.1 (17.6)	51.2 (19.9)	70.2 (19.1)	71.2 (18.5)	70.0 (17.3)	67.3 (15.2)
		77.1%	78.8%	108%	111%	108%	104%
Fat (g)	30	48.8 (29.8)	42.5 (21.0)	49.6 (26.7)	42.8 (20.8)	42.5 (23.1)	42.1 (20.4)
		163%	142%	165%	143%	141%	140%
Energy (kcal)	2,175	1,715 (567)	1,610 (494)	2,008 (600)	1,988 (417)	1,920 (283)	1,890 (336)
		78.8%	74.1%	92.3%	92.3%	88.4%	87.0%
Calcium (mg)	1,000	472 (230)	533 (299)	724 (320)	646 (291)	683 (269)*	584 (265)
		47.2%	53.3%	72.4%	64.6%	68.3%	58.4%
Iron (mg)	38	15.1 (6.1)	16.7 (9.1)	28.3 (6.5)†	22.3 (7.2)	28.4 (6.0)†	20.7 (4.5)
		39.7%	43.8%	74.5%	58.7%	74.8%	54.6%
β-carotene (ug)	2,400	288 (400)	393 (464)	1,893 *077*)†	438 (467)	1,880 (389)†	380 (406)
		12.0%	16.4%	78.9%	18.2%	78.3%	15.8%
Folate (ug)	400	192 (104)	173 (91)	244 (80)	223 (73)	241 (69)*	216 (64)
		48.0%	43.3%	61.0%	55.8%	60.2%	53.9%
Vitamin C (mg)	40	56.8 (75.0)	57.0 (56.0)	43.4 (53.5)	48.2 (56.4)	47.4 (67.0)	50.4 (66.0)
		142%	142%	108%	121%	119%	126%

*Evidence of difference between means comparing lcRTE with sRTE, p=0.07;

†Evidence of difference between means comparing lcRTE with sRTE, p<0.001;

‡Recommended Daily Allowance (RDA) for Indians (18)

**Table 4.** Maternal and infant outcomes by study group

Parameter		lcRTE (N=51)		sRTE (N=54)	Mean difference between groups (95% CI)
Maternal haemoglobin (g/dL)^a^	n	Mean (SD)	n	Mean (SD)	
14-16 weeks	50	8.83 (1.77)	53	8.38 (1.41)	0.45 (-0.18, 1.08), p=0.16^b^
35-36 weeks	45	8.99 (1.58)	47	7.75 (1.16)	1.24 (0.66, 1.82), p<0.001^b^
		Mean change (95% CI)^c^		Mean change (95% CI)^c^	
	45	0.27 (0.08, 0.46), p=0.006	47	−0.59 (−0.82, −0.35), p<0.001	0.94 (0.68, 1.20), p<0.001^d^
					
Maternal weight gain (kg)	n	Mean gain (95% CI)^e^	n	Mean gain (95% CI)^e^	
During 2nd trimester	46	2.60 (2.28, 2.92), p<0.001	51	2.41 (2.03, 2.78), p<0.001	0.20 (-0.29, 0.69), p=0.43^b^
During 3rd trimester	46	3.82 (3.35, 4.28), p<0.001	51	3.34 (3.02, 3.66), p<0.001	0.48 (-0.07, 1.02), p=0.09^b^
During 2nd and 3rd trimester	46	6.42 (5.88, 6.96), p<0.001	51	5.75 (5.27, 6.22), p<0.001	0.67 (-0.03, 1.38), p=0.06^b^
					
Abortion/miscarriage/stillbirth		n (%)		n (%)	
		6 (11.8%)		3 (5.9%)	p=0.49^f^
					
Live infant's birthweight (g)	n	Mean (SD)	n	Mean (SD)	
	45	2,695 (325)	51	2,545 (297)	150.5 (24.3, 276.6), p=0.02^b^
					
Live infant's gestational age (weeks)		n (%)		%	
≤37		6 (13.3%)		8 (15.7%)	p=0.74^f^
>37		39 (86.7%)		43 (84.3 %)	

^a^One woman in each group would not give blood for haemoglobin measurement at 14-16 weeks; at 35-36 weeks, haemoglobin was measured for 45 women in the lcRTE group and 47 women in the sRTE group;

^b^Student's *t-*test comparing Hb concentrations, weight gain, and infant's birthweight between groups;

^c^Student's paired *t-*test comparing the 3rd vs the 2nd trimester Hb concentrations within groups;

^d^Mean difference comparing lcRTE vs sRTE snack with Hb at 35-36 weeks as outcome, adjusted for Hb at 14-16 weeks (using a regression model);

^e^Student's *t-*test comparing weight at the end of the 2nd trimester with weight at beginning of the 2nd trimester, weight at the end of the 3rd trimester with weight at beginning of the 3rd trimester, and weight at the end of 3rd trimester with weight at the beginning of 2nd trimester;

^f^Fisher's exact test comparing proportions between groups

### Weight gain during pregnancy

The mean (SD) height of the subjects was 150.9 (5.8) cm and 152.2 (5.2) cm in the lcRTE and sRTE groups respectively; mean (SD) weight was 43.0 (6.3) kg and 42.9 (5.4) kg. Mean weight did not differ between vegetarian vs non-vegetarian women. There was a slightly higher overall weight gain in the lcRTE group (6.42 kg) compared to the sRTE group (5.75 kg) (Table 4). In a multivariable linear regression model among mothers with infants born alive, adjusting for mother's age, vegetarianism, education, baseline weight, infant's sex, and gestational age (>37 vs ≤37 weeks), lcRTE snacks increased the mother's weight by 0.62 kg (95% CI −0.07 to 1.32; p=0.08) compared to sRTE snacks. In this model, vegetarianism was independently associated with lower weight gain (-0.73 kg, 95% CI −1.46 to 0.00; p=0.05) and education vs no education with higher weight gain (0.75 kg, 95% CI 0.00-1.51; p=0.05).

### Pregnancy outcomes, gestational age, and birthweight

There were 2 stillbirths (1 following a fall, 1 following a severe gastrointestinal infection) and 4 abortions/miscarriages in the lcRTE group and 3 abortions/miscarriages in the sRTE group. There was no difference in the proportion of babies born alive before 37 weeks in the sRTE group (15.7%, 8/51) compared to the lcRTE group (13.3%, 6/45) (Table 4). The mean birthweight of infants born alive were lower in the sRTE group than in the lcRTE group by 151 g (95% CI 24-277; p=0.02). The proportions of babies born alive weighing <2,500 g in the lcRTE and sRTE groups were 22.2% (10/45) and 39.2% (20/51) respectively, although this difference was supported by only marginal statistical evidence (p=0.07 in chi-square test). In a multivariable linear regression model among infants born alive, adjusting for mother's age, body mass index in early 2nd trimester, vegetarianism, education, child's sex, and gestational age, lcRTE snacks increased infants’ birthweight by 116.5 g (95% CI −5.6 to 238.5; p=0.06) compared to sRTE snacks. In this model, gestational age >37 vs ≤37 weeks was associated with higher birthweight (mean difference 203.2 g, 95% CI 33.8-372; p=0.02) as was the mother's body mass index [mean difference 38.7 g, 95% CI 12.6-64.8 per unit (kg/m^2^) increase in body mass index].

## DISCUSSION

We have shown that fortification of a daily RTE protein/calorie snack with leaf concentrate has a beneficial effect on maternal haemoglobin concentrations and infant's birthweight. Our study is the first randomized controlled trial of leaf concentrate as a supplement during pregnancy. It provides evidence for the effectiveness of leaf concentrate as a food-based approach to combating micronutrient deficiencies in pregnancy and is another step towards substantiating the predominantly anecdotal evidence which has been reported over the past few years by advocates of leaf concentrate (14,19).

Our findings are of particular importance to public health campaigns that aim to combat maternal anaemia and low birthweight, given the need to find food-based supplements that are palatable and without side-effects and which have the potential for local production (11). Leaf concentrate, which is obtained after near-total exclusion of fibres and phytates (11), is likely to be a particularly acceptable food-based supplement in our study population, given that half of the women were vegetarian, and vegetables and pulses were a dietary staple. The provision of a snack that is consumed as a supplement rather than a substitute may be an important factor in improving maternal nutritional status (20).

In addition to iron and folic acid, 7 g of leaf concentrate contains nutritionally beneficial amounts of β-carotene (47% of RDA as retinol, 6:1 equivalence), vitamin E (40% of RDA), calcium (23% of RDA), and copper (8% of RDA). There is some evidence that multiple micronutrient supplementation as an adjunct to iron and folic acid supplements can reduce the problem of being small-for-gestational age and low birthweights (21-23), although effects on birthweight have tended to be smaller than the effect that we found for leaf concentrate, even after we adjusted our model for potential confounders. In our study setting, iron and folic acid supplements were not available through the ICDS programme. Hence, the LC-fortified snack was the only supplemental source of these micronutrients. Women in the control arms of other studies have usually been given iron and folic acid supplements. This difference in study design may account for the larger effect of micronutrient supplementation on infant's birthweight that we observed with other studies, although we note that the confidence interval for the effect of LC included null after adjustment for multiple covariates.

Early and late neonatal mortality does not appear to be directly reduced by multiple micronutrient supplementation (24) but low birthweight has been associated with worse outcomes among babies born in poor urban (25) and rural (26) districts of India. Copper has a role in haematopoiesis (27), and vitamin A and beta-carotene have been shown to improve iron absorption, possibly by preventing the inhibitory effects of phytates and polyphenols which are present in the usual diet in this population (28-30) and/or the inhibitory effects of inflammation (31). Regardless of these possible synergies, it is indisputable that pregnant women would benefit from the correction of other prevalent nutritional deficiencies (32), particularly of vitamin A (33) and calcium (34). Night blindness, a sign of vitamin A deficiency, was reported by 4.8% (5/105) of the women in our study at initial assessment (described as the inability to see when one steps out of the light and into the darkness, known locally as *Rataundhi*). No adverse effects of LC were reported. Indeed, the absence of side-effects may address the problem of low adherence to anaemia prevention campaigns based on iron tablets.

Pregnant women in this community followed a practice of eating only lunch (10:00 am) and an evening meal (8:00 pm), with no other food consumed between these two meals, other than tea. Although adjustments in basal metabolism and physical activity can offset the additional energy demands of gestation (35), suboptimal energy intake has been associated with infant's low birthweight (36). The protein and calorie contents of the snacks ameliorated protein and energy deficiencies in the diet, and women in both the groups gained weight to an equal degree, although, at approximately 6 kg, these gains were well below the 12.5-18.0 kg range recommended for women with low BMI (37). Both LC and soya are sources of complete protein, and their inclusion in the RTE snack would improve the suboptimal protein/energy ratio of the wheat- and rice-based predominantly vegetarian diet prevalent in poor Indian communities (38).

### Limitations

One limitation of our study was the unmeasured effect of inflammation (39). We have no reason to suspect that inflammation would be unequally distributed between the two groups but repeated measurements might have suggested a mechanism for the protective effect of LC against declining maternal haemoglobin concentrations. Losses to follow-up were small in both the arms but consumption of the RTE snacks could not be rigorously verified.

Our analysis follows the intention-to-treat approach that correctly measures the relative effectiveness of an intervention in a randomized controlled trial. Baseline haemoglobin was slightly higher in the group that received the LC-fortified supplement but we still found a substantial effect of LC compared to the standard snack after adjustment for baseline haemoglobin and other potential confounders.

### Conclusions

We have demonstrated, in a small trial, that leaf concentrate as an adjunct to protein/calorie supplements is effective in preventing declining maternal haemoglobin concentrations and in increasing infant's birthweight. The effects of LC in our study were larger than those reported from other micronutritional interventions in undernourished pregnant women, and it remains to replicate our findings in larger randomized controlled trials among diverse target populations. LC was available for this study because it was being produced for two LC-based feeding programmes. Further research into the economic and social aspects of various types of leaf concentrate production in India is needed to provide an evidence-base for sustainable local production.
